# Dengue virus infection and potential association with acute lymphoblastic leukemia in Egypt: a pilot study

**DOI:** 10.1186/s13027-026-00738-7

**Published:** 2026-03-10

**Authors:** Lamiaa Fadel Alkilany, Shaza Fadel Alkilany, Azza Shibl, Doaa A. Mohammed, Mayada Fawzy Sedik, Hyam H. Mahran, Yomna R. Mahboub, Hany K. Soliman, Aliaa M. A. Ghandour

**Affiliations:** 1 Virology and Immunology Unit, Cancer Biology Department, South Egypt Cancer Institute, Assiut University, Assiut, 71515 Egypt; 2 Public Health and Community Medicine Department, Faculty of Medicine, Minia University, Minia, Egypt; 3 Pediatric Oncology and Hematological Malignancy Department South Egypt Cancer Institute, Assiut University, Assiut, Egypt; 4 Clinical Pathology Department, South Egypt Cancer Institute, Assiut University, Assiut, Egypt; 5 Medical Oncology and Malignant Hematology Department, South Egypt Cancer Institute, Assiut University, Assiut, Egypt; 6 Pediatric Department, Assuit University Hospitals, Assiut, Egypt; 7 Clinical Hematology and Internal Medicine Department, Assuit University Hospitals, Assiut, Egypt; 8 Virology and Immunology Unit, Cancer Biology Department, National Cancer Institute, Cairo University, Cairo, Egypt; 9 Medical Microbiology and Immunology Department, Faculty of Medicine, Assuit University, Assiut, Egypt

**Keywords:** Dengue, Lymphoblastic, Leukemia, BCR-ABL, Fusion, Gene, Pilot

## Abstract

**Objectives:**

Dengue fever is arthropod born disease transmitted by the bite of a mosquito called Aedes aegypti. Some researchers have suggested a link between previous dengue infection and leukemia. The aim of this pilot study is to determine the frequency of dengue infection in acute lymphoblastic leukemia patients (ALL) compared to age and sex matched controls and to find the impact of this infection on the disease outcome.

**Patients and methods:**

This is a single center pilot study that included two groups, 30 newly diagnosed pediatric ALL patients and 22 newly diagnosed adult ALL patients that were compared with 50 age and sex matched healthy controls (25 for each group) and 8 pediatric immunosuppressed controls. ELISA was used to detect IgM and IgG antibodies in serum samples at presentation and clinical and laboratory data at admission were collected. Positive and negative dengue patients were followed up for a minimum of six months (hybrid case control –cohort study).

**Results:**

Among pediatric patients who were evaluated for dengue infection by the serological tests, 46.7% (14/30), 8% (2/25) and 0% (0/8) tested positive for IgG in patients, immuno-competent and immunocompromised controls respectively (p value 0.002). There was a significant association between acute dengue infection and BCR-ABL fusion oncogenic gene only in pediatric patients. No statistically significant difference in short term outcomes was observed.

**Conclusion:**

This pilot study provides preliminary data to guide large scale research on the role of dengue infection as a predisposing or exacerbating factor in genetically susceptible children.

## Introduction

Dengue fever is a mosquito-born, acute febrile illness caused by dengue virus. The container-breeding *Aedes aegypti* is the most typical dengue virus vector and often found in urban areas [1]. Dengue infection is usually presented by non-specific symptoms like fever, rash, nausea, muscle pain and swollen glands, but rarely presented with life threatening severe manifestations like dengue hemorrhagic fever [2]. 

The association between hematological malignancies and dengue infection remains unclear with limited literature on the topic. The only notable study is a nationwide population-based cohort study conducted in Taiwan in 2020 which analyzed the National Health Insurance Research Databases. This study identified laboratory-confirmed dengue patients between 2002 and 2011 and found that dengue virus infection was significantly associated with a higher risk of leukemia 3 and 6 years after infection, but was no such association was observed with other cancers [3].

A study in 2024 reported two cases of acute leukemia following dengue infection in a recent outbreak in Nepal which suggests a possible association of acute leukemia with dengue infection [4]. 

Acute lymphoblastic leukemia (ALL) is the most common malignancy in pediatrics [5]. 

The only virus that was linked to human leukemia is T-cell lymphoma/leukemia virus-1 (HTLV-I&II) [6].

In Egypt, The situation regarding dengue virus infection is not well documented due to lack of screening and the fact that the majority of dengue cases are asymptomatic leading to underreporting [7].

A 2020 study found that the seroprevalence of dengue fever in humans in Assuit and Sohag governorates in Egypt was 12.09% [8].

To date, no studies have reported the frequency of this virus in ALL patients or its association with clinical and laboratory characteristics nor its potential impact on the disease outcome.

## Methodology

### Study subjects

This is a pilot study that was designed as a hybrid case-control and cohort study.

#### The case –control element of the study design

It included two patient groups; pediatric group of 30 newly diagnosed ALL pediatric patients admitted to Pediatric Oncology and Hematological Malignancies Department and 22 adult ALL patients admitted to Medical Oncology Department at South Egypt Cancer Institute in the period from 1 July 2024 to 31 December 2024 and included 50 age and sex matched healthy controls (25 for each group) and 8 immunosuppressed pediatric controls from other malignant or non-malignant conditions (3 patients diagnosed with acute myeloid leukemia, 3 HLH, 2 aplastic anemia). Relapsed leukemic patients, those with previous treatment before presentation, second malignancy after chemotherapy or radiotherapy and leukemia not confirmed by flow cytometry were excluded. The study was approved by Institutional Review Board of South Egypt Cancer Institute Assiut University Egypt (approval number 770 SECI-IRB, IORG0006563).

### Materials and methods

1. Clinically suspected case of acute leukemia underwent laboratory investigations: Complete blood count (CBC) with peripheral blasts detection that was confirmed by Bone Marrow Aspirate (BMA). Lineage identification was performed by flow cytometry. Routine cytogenetic tests by FISH technique (fluorescence in situ hybridization) were done for diagnosing a genetic abnormality; BCR-ABL fusion gene (breakpoint cluster region gene fused with the Abelson (ABL) proto-oncogene).
2. According to the previous data, the patients were risk stratified and chemotherapy regimens were started. Pediatric patients were treated according to Total XV protocol of SJCRH (ST Jude Children’s Research Hospital) [9], adult patients received Hoelzer protocol [10]. The treatment response was assessed by CBC, BMA and cerebrospinal fluid (CSF).
3. Sampling: One ml of blood was withdrawn from patients and controls. The sample was centrifuged and the serum was preserved in -70 C freezer.
4. Serology testing: The serum levels of specific dengue IgG and IgM were measured using Dengue virus _F_-IgG and Dengue virus _F_-IgM ELISA kits (Native Antigen, LGC Clinical Diagnostics, Co.) which detect dengue antibodies in the serum. The color change was measured by spectrophotometry at a wavelength 450 nm using SPECTROstar Nano absorbance plate reader (BMG LABTECH, Germany). The cut-off point was calculated according to the manufacturer’s instructions as follows: average optical density of cutoff controls and equaled 0.15 in our assay.
5. Results are expressed in arbitrary units [U].
  $$\frac{\text{Sample (mean) absorbance value} \times \mathrm{10}}{\text{Cut - off}} = [\text{arbitrary units} = \mathrm{U}]$$  

- #Results are interpreted as follows
- > 10 positive
- < 9 negative
- 9–11(repeated)

Acute dengue infection was diagnosed by detection of specific IgM antibodies with or without dengue IgG antibodies and past infection was diagnosed by detection of dengue IgG antibodies with no detection of dengue IgM antibodies.

#### The cohort element of the study design

The patient cohort included 52 patients (30 newly diagnosed pediatric ALL patients and 22 newly diagnosed adult ALL patients) and were classified into two groups according to dengue infection serology results (IgM positive and IgM negative groups indicating recent exposure status) and (IgG positive and IgG negative groups indicating prior exposure status) and were followed up for a minimum of 6 months to determine the prognosis of dengue infected patients including death and relapse compared to dengue negative patients. Relapse was defined as recurrence of disease in BM, CSF or other site after achieving complete remission. Complete remission (CR) is the absence of leukemic blasts in peripheral blood and CSF and less than 5% blasts on BMA smears with no evidence of extra medullary site invasion and minimal residual disease (MRD) of less than 0.01% [11].

### Statistical analysis

The analysis of the data was carried out using the IBM SPSS 27.0 statistical package software (IBM; Armonk, New York, USA). Data were expressed as median and interquartile range (IQR) for non-parametric data, in addition to both number and percentage for categorized data.

The Mann-Whitney U for quantitative non-parametric data was used for comparison between two independent groups. Kruskal-Wallis (KW) statistical test was used for comparison between independent groups for non-parametric data followed by Dunn’s post-hoc test to assess intergroup differences. The Chi-square test or Fisher’s exact test were used to compare categorical variables. Survival curves were plotted using Kaplan-Meier method and the significance of difference between survival curves was determined using log-rank ratio. Overall survival (OS) was defined as the time from diagnosis to death of any cause. Relapse free survival (RFS) was defined as the time from complete remission to recurrence of leukemia. A p-value less than 0.05 was considered significant.

## Results

This study included 30 cases of pediatric ALL patients with median age 6 years (IQR, 4.5-9) and 25 age and sex matched pediatric controls with median age 7 years (IQR, 5–9). The study also included 22 adult ALL patients with median age 35 years (IQR, 32–44) with 25 age and sex matched healthy controls with median age 34 years (IQR, 33–43). Another eight immunosuppressed pediatric patients were included in a separate control group with median age 8 years (IQR, 6.5–9.5) as seen in Table 1.

**Table 1:** Demographics of patients and controls in pediatric and adult groups

Pediatric					
	Patients	Control	Immuno-suppressive controls (IS)	*p* value (cases vs. controls)	*p* value (cases vs. IS controls)
	(no = 30)	(no = 25)	(no = 8)		
**Age (y)**					
Median (IQR)	6 (4.5-9)	7 (5–9)	8 (6.5–9.5)	0.381	0.314
**Sex**					
Male	21 (70.0%)	15 (60.0%)	5 (62.5%)	0.437	0.685
Female	9 (30.0%)	10 (40.0%)	3 (37.5%)		

Adults					
	Patients	Control		p value (cases vs. controls)	
	(no = 22)	(no = 25)			
**Age (y)**					
Median (IQR)	35 (32–44)	34 (33–43)		0.983	
**Sex**					
Male	16 (72.7%)	14 (56.0%)		0.234	
Female	6 (27.3%)	11 (44.0%)			

IQR, interquartile range

In pediatric group, BCR-ABL cytogenetic abnormality represented 13.3% (4 cases).

In adult cases, Patients tested positive for BCR-ABL fusion gene represented 22.7% of cases. See Table 2.

**Table 2:** Disease characteristics of pediatric and adult patients

	Pediatric	Adult
	(no = 30)	(no = 22)
**Subtype**		
B-cell	22 (73.3%)	19 (86.4%)
T-cell	8 (26.7%)	3 (13.6%)
**Risk**		
Standard risk	16 (53.3%)	17 (77.3%)
High risk	14 (46.7%)	5 (22.7%)
**WBCs (x10^3 /µL)**		
Median (IQR)	33 (11.7–112)	44 (36–55)
**Hb (g/d)**		
Median (IQR)	7.5 (6–8)	6.7 (6-7.5)
**Platelet (x10^3 /µL)**		
Median (IQR)	44 (24–93)	51 (33–60)
**BCR-ABL fusion gene abnormality**		
Negative	26 (86.7%)	17 (77.3%)
Positive	4 (13.3%)	5 (22.7%)

Among 30 pediatric patients who were evaluated for dengue infection by the serological tests, (46.7%, 8%) tested positive for IgG in patients and immuno-competent controls respectively and no IgG positive cases in immunosuppressed controls. The difference was significant between pediatric patients and either healthy control or immunosuppressed control (0.002 & 0.015 respectively).

Regarding IgM, 26.7% of patients (8 patients) were tested positive for dengue specific IgM which is significantly different from its percentage in immuno-competent controls (1/25) (4.0%) (*P* = 0.031).

Among adult patients, 7 adult cases (31.8%) and 12 (48%) controls were positive for IgG while none of the cases were positive for IgM. See Table 3.

**Table 3:** Serological diagnosis of dengue virus infection in patients and controls

Pediatrics					
	Case	Control	Immuno-suppressive controls	*p* value (cases vs. control)	*p* value (cases vs. IS control)
	(no = 30)	(no = 25)	(no = 8)		
**Dengue IgG**					
Negative	16 (53.3%)	23 (92.0%)	8 (100.0%)	0.002*	0.015*
Positive	14 (46.7%)	2 (8.0%)	0 (0.0%)		
**Dengue IgM**					
Negative	22 (73.3%)	24 (96.0%)	8 (100.0%)	0.031*	> 0.99
Positive	8 (26.7%)	1 (4.0%)	0 (0.0%)		
**Dengue infection**					
Acute	8 (26.7%)	1 (4.0%) ^#^	0 (0.0%)	0.003*	> 0.99
Past	7 (23.3%)	1 (4.0%) ^#^	0 (0.0%)		
No infection	15 (50.0%)	23 (92.0%) ^#^	8 (100.0%)		

Adults					
	Case	Control		p value (cases vs. control)	
	(no = 22)	(no = 25)			
**Dengue IgG**					
Negative	15 (68.2%)	13 (52.0%)		0.259	
Positive	7 (31.8%)	12 (48.0%)			
**Dengue IgM**					
Negative	22 (100.0%)	25 (100.0%)		….	
Positive	0 (0.0%)	0 (0.0%)			
**Dengue infection**					
Acute	0 (0.0%)	0 (0.0%)		0.259	
Past	7 (31.8%)	12 (48.0%)			
No infection	15 (68.2%)	13 (52.0%)			

^#^Significant difference between column proportions (compared using z test)

In pediatric patients, there was a significant decrease in platelet count in acute dengue infection compared to past infection (p value 0.007*). There was a significant association between acute dengue infection and BCR–ABL fusion gene (Philadelphia chromosome) (p value 0.048) as seen in Table 4.

**Table 4:** Demographical and disease characteristics of acute, past and negative infection of dengue virus in pediatric cases

Pediatric cases						
	Dengue infection					
	Acute	Past	Negative	*p* value		
	(no = 8)	(no = 7)	(no = 15)			
**Age (y)**						
Median (IQR)	6 (4–10)	7.5 (5.5–8.5)	7 (5–9)	0.568		
**Sex**						
Male	6 (75.0%)	4 (57.1%)	11 (73.3%)	0.764		
Female	2 (25.0%)	3 (42.9%)	4 (26.7%)			
**Subtype**						
B-cell	7 (87.5%)	5 (71.4%)	10 (66.7%)	0.579		
T-cell	1 (12.5%)	2 (28.6%)	5 (33.3%)			
**Risk**						
Standard risk	4 (50.0%)	3 (0.42.9%)	9 (60%)	0.695		
High risk	4 (50.0%)	4 (57.1%)	6 (40%)			
**WBCs (x10^3 /µL)** Median (IQR)	54 (37.5–156)	32 (9.7–116)	23 (11–52)	0.293		
**Hb (g/d)** Median (IQR)	7.5 (6–8)	7.5 (7–8)	7 (6–8)	0.718		
**Platelet (x10^3 /µL)** Median (IQR)	24.5 (14.5–42)	90 (77–128)	44 (23–115)	0.025*		
				acute vs. past	acute vs. negative	past vs. negative
				0.007*	0.099	0.137

	Acute	Past and negative infection				
	*n* = 8	*n* = 22				
**BCR-ABL fusion gene abnormality**						
Negative	5 (62.5%)	21 (95.5%)		0.048*		
Positive	3 (37.5%)	1 (4.5%)				

In adults there were no acute infections. There was no significant difference in platelet count or cytogenetic abnormality at the time of diagnosis between positive and negative dengue infection as seen in Table 5.

**Table 5:** Demographical and disease characteristics of IgG positive and IgG negative adult cases

	Adult		
	Dengue infection		*p* value
	IgG positive	IgG negative	
	(no = 7)	(no = 15)	
**Age (y)**			
Median (IQR)	36 (32–45)	34.5 (32–44)	0.777
**Sex**			
Male	6 (85.7%)	10 (66.7%)	0.616
Female	1 (14.3%)	5 (33.3%)	
**Subtype**			
B-cell	5 (71.4%)	14 (93.3%)	0.227
T-cell	2 (28.6%)	1 (6.7%)	
**Risk**			
Standard risk	6 (85.7%)	11 (73.3%)	> 0.99
High risk	1 (14.3%)	4 (26.7%)	
**WBCs (x10^3 /µL)**			
Median (IQR)	50 (33–70)	44 (39–50)	0.620
**Hb (g/d)**			
Median (IQR)	6.5 (6-7.7)	7 (6-7.5)	0.775
**Platelet (x10^3 /µL)**			
Medan (IQR)	55 (33–60)	45 (31–60)	0.944
**BCR-ABL fusion gene abnormality**			
Negative	6 (85.7%)	11 (73.3%)	> 0.99
Positive	1 (14.3%)	4 (26.7%)	

### Survival analysis by dengue infection status in adult and pediatric ALL patients

The follow up period of patient cohort ranged from 2 months to 12 months in adult group and 3 months to 12.2 months in pediatric group. The median follow up time was 9.6 months in adult group and 7.6 months in pediatric group.

**Fig. 1:**
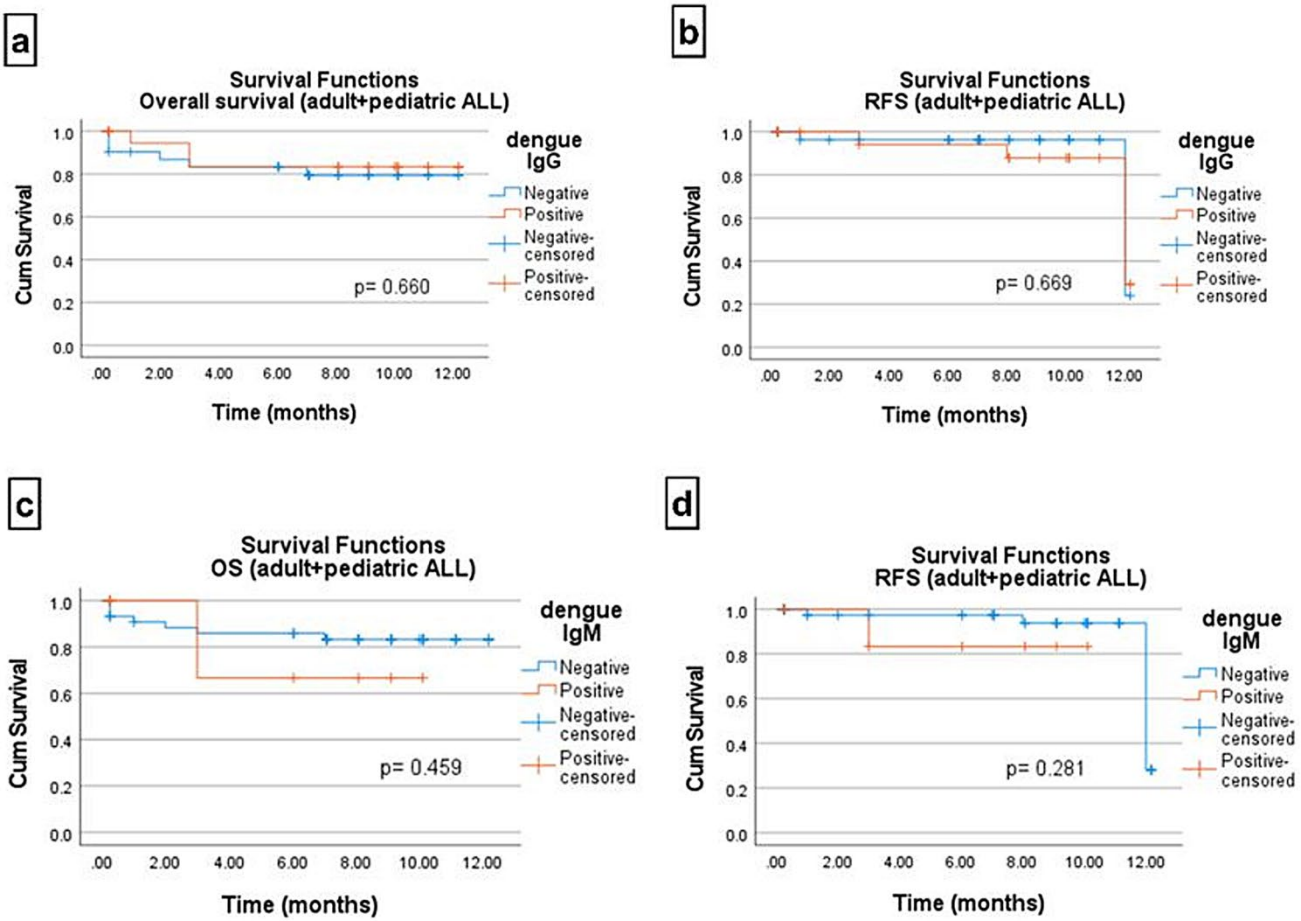
(**a**) Overall Survival (OS) in acute lymphoblastic leukemia adult and pediatric patients stratified by dengue virus seropositivity (IgG). (**b**) Relapse-free survival (RFS) in acute lymphoblastic leukemia adult and pediatric patients stratified by dengue virus seropositivity (IgG). (**c**) Overall Survival (OS) in acute lymphoblastic leukemia adult and pediatric patients stratified by the presence of acute dengue virus infection (IgM positivity). (**d**) Relapse-free survival (RFS) in acute lymphoblastic leukemia adult and pediatric patients stratified by the presence of acute dengue virus infection (IgM positivity)

One year OS for all included patients did not significantly differ between IgG positive (83.3%) with mean survival time (10.5 m ± 0.9 SE) and negative cases (79.4%) with mean survival time (10.1 m ± 0.8 SE) (p value 0.6) as in figure (1). One year RFS was 29.3% for IgG-positive patients versus 24.1% for IgG negative (P value 0.669).

Cases positive for IgM did not completed the one year follow up. Six month OS was 66.7% for IgM positive cases with mean survival time (7.7 m ± 1.4 SE) and 85.9% for IgM negative cases with mean survival time (10.5 m ± 0.6 SE) with no significant difference between them (p value 0.459). Six month RFS for IgM positive cases was 83.3% and 97.4% for IgM negative cases with no significant difference between them (p value 0.281). See Fig. 1.

## Discussion

Dengue virus is one of the important viruses in research due to its global public health impact but it is rarely diagnosed in clinical settings because of its nonspecific presentation in most cases [12]. It’s even more difficult to be diagnosed in leukemia patients as the clinical symptoms and signs even severe ones like bleeding tendency are obscured by the underlying leukemia. To our knowledge, this is the first study that documented dengue seroprevalence in leukemia patients.

In this study, past infection was diagnosed by detection of IgG antibodies and acute dengue infection by IgM antibody, most of pediatric leukemia patients were diagnosed and confirmed by flow cytometry two weeks or a month after the beginning of symptoms. So, diagnosis of acute dengue infection by NS1 (nonstructural protein1) test may be inappropriate as it typically remains in the serum for only 5 to 6 days after the onset of fever while IgM antibodies can persist for about 6 weeks [13].

The prevalence of Dengue IgG in healthy pediatric controls was 8%, compared to 48% in healthy adults. This difference can be attributed to the increased risk of exposure with age. A previous study conducted in 2019 documented a dengue seroprevalence of 12.09% in Assuit and Sohag governorates in Egypt [14].

The increase in seroprevalence over the 6-year period can be attributed to the influx of Sudanese refugees into Egypt following the 2023 Sudanese civil war. Dengue fever is highly endemic in Sudan with multiple outbreaks occurring during the war due to partial collapse of the health care system [15].

Acute dengue fever diagnosed by specific IgM was not found among adult controls but represented 4% of pediatric controls (1 case). Another study found that about one third of dengue suspected cases admitted to fever hospitals (33.3%) were positive for dengue virus by rapid dipstick immuno-chromatographic analysis. This high rate was attributed to the fact that the detection occurred among the suspected cases during an outbreak rather than the general population [16].

As seen in Table 3, a significant difference in the seroprevalence of dengue virus (IgG) was found in early diagnosed leukemic children compared to healthy controls, and the percentage of pediatric patients diagnosed with acute dengue fever at presentation was significantly higher than healthy controls (p value 0.031). This can be explained by immune suppression in leukemia patients caused by destruction of the immune cells by leukemic blasts, The percentage was also significantly higher than immune-suppressed controls that were included during the same period and presented with various malignant and non-malignant conditions. It appears that dengue virus infection is significantly associated with ALL regardless of immune status. Additionally, there was a significant increase in the percentage of pediatric leukemic patients with past exposure to dengue virus than in healthy controls (p 0.003 value).

In adult patients, there was no significant difference in the IgG prevalence between cases and controls (p value 0. 259). Exposure to dengue virus was notably high in the adult control group. Previous exposure to dengue provides lifelong immunity against infection by the same serotype as well as short term immunity against other serotypes [17], This could explain the absence of acute dengue infection in adult patients.

A previous study conducted in Taiwan in 2020 was the only one to document an increased risk of leukemia following dengue virus infection with the highest risk observed 3–6 years after infection. However, they did not link this risk to a certain type of leukemia [3]. In contrast, this study focused exclusively on pediatric ALL patients. Roughly It is estimated that 15–20% of human cancers are linked to viral infections [18]. Pediatric ALL has long been suspected by many scientists to have a viral origin [19]. Leukemia in various animal species like cats, cattle, chickens and mice has been shown to be associated with oncogenic viruses [20]. It is possible that human leukemia could also be induced by a similar transforming virus, although to date, no acute transforming viruses have been reported in humans.”

In the current study, there was a significant association between acute dengue infection and BCR–ABL fusion gene. This oncogene is generated by the Philadelphia chromosome translocation leading to increased activity of ABL tyrosine kinase [21]. The small sample size of cases associated with this abnormality limits generalization of this finding.

Studies on seasonal variation in ALL onsets have supported the infection theory of pediatric leukemia [19]. It is worth noting that dengue virus also follows seasonal patterns as well. Dengue virus season comes with higher Aedes mosquito activity during warm temperatures and rainy seasons [22].

Because of the observation that leukemia is more common in developed countries, the researchers have embraced the hygiene hypothesis which suggests that delayed exposure to infections increased risk of leukemia due to delayed training of the immune system.

The infection theory does not blame the infectious agent by itself but rather the desregulated immune response to infection [23]. It has also been suggested that infection may act as an exacerbating factor or secondary event in an individual who is already susceptible or initiated [24].

Dengue virus can lead to direct infection of bone marrow, which in some cases results in aplastic anemia [25], many case reports have documented occurrence of ALL following a period of bone marrow aplasia [26, 27]. One study found that aplastic anemia patients go on to develop secondary myelodysplastic syndrome or acute myeloid leukemia by 10 years of follow-up [28].

In this study, there was a significant drop in platelet count in acute dengue infection compared to past infection in pediatric cases (p value 0.007). Severe dengue infections are sometimes associated with severe thrombocytopenia that may lead to bleeding tendency. Thrombocytopenia may result from bone marrow suppression, immune mediated destruction or induced apoptosis [29]. This finding may be masked by thrombocytopenia caused by leukemia itself.

A main limitation in this study is the lack of NS1 antigen testing or PCR confirmatory testing to rule out cross reactivity with other flaviviruses. The use of the confirmatory tests posed several challenges, NS1 antigen testing sensitivity drop sharply after 5 days of beginning of symptoms and viremia usually clears so false negative PCR becomes common. This infection is self-limiting even in leukemia patients [30] who are usually presented 2 weeks from the beginning of symptoms because of non-specific presentation and delayed access to care.

Another limitation of this study is the heterogenicity of the control group as the immune status may vary widely introducing residual confounders but it is advised in future studies with large sample size to allow generalizability with careful statistical adjustment (e.g.,stratification, covariates).

This is a hypothesis generating pilot study that does not aim to prove causation but the results can be used to formulate testable hypothesis for future large cohort studies that minimize these limitations as possible. and confirm the association between dengue infection and the oncogenic BCR-ABL fusion gene. Thia association may indicate that dengue is a predisposing or exacerbating factor for genetically susceptible individuals.

## Conclusion

There is a significantly high frequency of dengue virus infection in newly diagnosed ALL patients compared to both healthy and immunosuppressed controls only in pediatrics. Since association does not imply causation, further studies are needed to confirm the relationship between dengue infection and leukemia as well as between dengue infection and oncogenic abnormalities.

## Data Availability

The datasets used and/or analyzed during the current study are available from the corresponding author on reasonable request.

## Abbreviations

ALL: Acute lymphoblastic leukemia
BCR-ABL: Breakpoint cluster region gene fused with the Abelson
BMA: Bone marrow aspirate
CR: Complete remission
CSF: Cerebrospinal fluid
MRD: Minimal residual disease
NS1: Nonstructural protein
OS: Overall survival
SE: Standard error
RFS: Relapse free survival
SJCRH: ST Jude Children’s Research Hospital

## References

[1] Hasan S, et al. Dengue virus: A global human threat: review of literature. J Int Soc Prev Community Dentistry. 2016;6(1):1–6. 10.4103/2231-0762.175416 PMC4784057 27011925

[2] Htun TP, Xiong Z, Pang J. Clinical signs and symptoms associated with WHO severe dengue classification: a systematic review and meta-analysis. Emerg Microbes Infect. 2021;10(1):1116–28. 10.1080/22221751.2021.1935327 PMC8205005 34036893

[3] Chien YW, et al. Risk of leukemia after dengue virus infection: A Population-Based cohort study. Cancer Epidemiol Biomarkers Prev. 2020;29(3):558–64. 32051189 10.1158/1055-9965.EPI-19-1214

[4] Agrawal A, et al. Acute leukaemia following dengue infection in Nepalese patients: a report of two cases*.* Case Rep Hematol. 2024;2024:8747138. 10.1155/2024/8747138 PMC11298969 39104430

[5] Ekpa QL, et al. A review of acute lymphocytic leukemia (ALL) in the pediatric population: evaluating current trends and changes in guidelines in the past decade. Cureus. 2023;15(12). 10.7759/cureus.49930 PMC10766210 38179374

[6] Satou Y, Matsuoka M. Virological and immunological mechanisms in the pathogenesis of human T-cell leukemia virus type 1. Rev Med Virol. 2013;23(5):269–80. 23606621 10.1002/rmv.1745

[7] De Santis O, Bouscaren N, Flahault A. Asymptomatic dengue infection rate: a systematic literature review. Heliyon. 2023;9(9). 10.1016/j.heliyon.2023.e20069 PMC10559824 37809992

[8] Hussen MO, Sayed ASM, Abushahba MFN. Sero-epidemiological study on dengue fever virus in humans and camels at upper Egypt. Vet World. 2020;13(12):2618–24. 33487979 10.14202/vetworld.2020.2618-2624 PMC7811540

[9] Pui C-H, et al. Treating childhood acute lymphoblastic leukemia without cranial irradiation. N Engl J Med. 2009;360(26):2730–41. 19553647 10.1056/NEJMoa0900386 PMC2754320

[10] Hoelzer D, et al. Intensified therapy in acute lymphoblastic and acute undifferentiated leukemia in adults*.* 1984. 6375764

[11] Yang EJ, et al. Treatment outcome of pediatric acute lymphoblastic leukemia in Yeungnam region: multicenter retrospective study of study alliance of Yeungnam pediatric Hematology-Oncology (SAYPH). Pediatr Hematol Oncol. 2018;35(4):276–87. 30633619 10.1080/08880018.2018.1483986

[12] Ulgheri FM, Bernardes BG, Lancellotti M. Decoding dengue: A global Perspective, History, Role, and challenges. Pathogens. 2025;14(9):954. 41011854 10.3390/pathogens14090954 PMC12472879

[13] Centers for Disease Control and Prevention. Serologic tests for dengue virus. https://www.cdc.gov/dengue/healthcare-providers/testing/serologic-tests.html. accessed Nov 1.

[14] Hussen MO, Sayed ASM, Abushahba MFN. Sero-epidemiological study on dengue fever virus in humans and camels at upper Egypt. Veterinary World. 2020;13(12):2618. 33487979 10.14202/vetworld.2020.2618-2624 PMC7811540

[15] Mahjaf GM, Abdelgader LMA, Abuzeid NMK. High Seroprevalence of dengue virus infection among febrile patients in Kassala state, Eastern sudan: a cross-sectional analysis. J Bacteriol Mycol Open Access. 2025;13(3):141–4.

[16] Gaber M, et al. Dengue fever as a reemerging disease in upper egypt: Diagnosis, vector surveillance and genetic diversity using RT-LAMP assay. PLoS ONE. 2022;17(5):e0265760. 35499983 10.1371/journal.pone.0265760 PMC9060354

[17] Rothman AL. Immunity to dengue virus: a Tale of original antigenic sin and tropical cytokine storms. Nat Rev Immunol. 2011;11(8):532–43. 21760609 10.1038/nri3014

[18] McLaughlin-Drubin ME. K. <>Munger 2008 Viruses associated with human cancer. Biochim Et Biophys Acta (BBA)-Molecular Basis Disease 1782 3 127–50. 10.1016/j.bbadis.2007.12.005 PMC2267909 18201576

[19] McNally RJQ, Eden TOB. An infectious aetiology for childhood acute leukaemia: a review of the evidence. Br J Haematol. 2004;127(3):243–63. 15491284 10.1111/j.1365-2141.2004.05166.x

[20] de Moura FBC. A systematic review on infectious agents inducing cancer in animals: What do we know? Comparat Transl Med. 2024;2(1).

[21] Sattlermc M, Griffin JD. Molecular mechanisms of transformation by the BCR-ABL oncogene. Semin Hematol. 2003;40:4–10. 10.1053/shem.2003.50034 12783368

[22] Liu Z, et al. The effect of temperature on dengue virus transmission by Aedes mosquitoes. Front Cell Infect Microbiol. 2023;13:1242173. 37808907 10.3389/fcimb.2023.1242173 PMC10552155

[23] Greaves M. The ‘delayed infection’ (aka ‘hygiene’) hypothesis for childhood leukaemia. The hygiene hypothesis and darwinian medicine. Birkhäuser Basel: Basel; 2009. pp. 239–55. G.A.W. Rook, Editor.

[24] Greaves M. A causal mechanism for childhood acute lymphoblastic leukaemia. Nat Rev Cancer. 2018;18(8):471–84. 29784935 10.1038/s41568-018-0015-6 PMC6986894

[25] Noisakran S, et al. Infection of bone marrow cells by dengue virus in vivo. Exp Hematol. 2012;40(3):250–9. 22193689 10.1016/j.exphem.2011.11.011 PMC3415316

[26] Matloub YH, et al. Severe aplastic anemia preceding acute lymphoblastic leukemia. Cancer. 1993;71(1):264–8. 8416724 10.1002/1097-0142(19930101)71:1<264::aid-cncr2820710140>3.0.co;2-8

[27] Naturinda E, et al. Transient bone marrow hypoplasia preceding T-Cell acute lymphoblastic leukemia: a case report. Afr Health Sci. 2021;21(2):683–6. 34795723 10.4314/ahs.v21i2.25 PMC8568206

[28] Sun L, Babushok DV. Secondary myelodysplastic syndrome and leukemia in acquired aplastic anemia and paroxysmal nocturnal hemoglobinuria*.* Blood. J Am Soc Hematol. 2020;136(1):36–49. 10.1182/blood.2019000940 PMC7332901 32430502

[29] Khazali AS, et al. Thrombocytopenia in dengue infection: mechanisms and a potential application. Expert Rev Mol Med. 2024;26:e26. 39397710 10.1017/erm.2024.18 PMC11488332

[30] Kini RG, Moras CB. The double jeopardy of leukemia and dengue: a report of three cases. J Appl Hematol. 2020;11(1).

